# Inhibitory effects of *Syzygium jambos* extract on biomarkers of endothelial cell activation

**DOI:** 10.1186/s12906-022-03572-7

**Published:** 2022-04-07

**Authors:** Yaritza Inostroza-Nieves, Shirley Valentin-Berrios, Christopher Vega, Gregory N. Prado, Claribel Luciano-Montalvo, José R. Romero, Alicia Rivera

**Affiliations:** 1 Division of Endocrinology, Diabetes and Hypertension, Department of Medicine, Brigham and Women’s Hospital, and Harvard Medical School, Boston, MA USA; 2 Department of Biochemistry and Pharmacology, San Juan Bautista School of Medicine, PO Box 4968, Caguas, PR 00726-4968 USA; 3 Department of Laboratory Medicine, Boston Children’s Hospital and Department of Pathology, Harvard Medical School, Boston, MA USA; 4 Department of Microbiology, San Juan Bautista School of Medicine, Caguas, PR USA; 5 Faculty of Science and Technology, Inter American University of Puerto Rico, Metropolitan Campus, San Juan, PR USA

**Keywords:** Endothelin-1, Interleukin-6, Protein disulfide isomerase, *Syzygium jambos*, Endothelial cells, Reactive oxygen species

## Abstract

**Background:**

Disordered endothelial cell activation plays an important role in the pathophysiology of atherosclerosis, cancer, sepsis, viral infections, and inflammatory responses. There is interest in developing novel therapeutics to regulate endothelial cell function in atherothrombotic, metabolic, vascular, and hematological diseases. Extracts from leaves of the *Syzygium jambos* (L.) Alston (*S. jambos*) trees have been proposed to treat cardiovascular diseases and diabetes through unclear mechanisms. We investigated the effects of the *S. jambos* extract on biomarkers of endothelial dysfunction and immune responses in the human endothelial cell line, EA.hy926.

**Methods:**

Leaves of *S. jambos* were collected, concocted and lyophilized. To study the effects of *S. jambos* on endothelial cell activation, we used the human endothelial cell line. IL-6 levels were measured using qPCR and ELISA. PDI activity was measured using Insulin Turbidity and Di-E-GSSG assays. CM-H2DCFDA was used to study ROS levels. Migration assay was used to study *S. jambos* effect on ex vivo human polymorphonuclear and human mononuclear cells.

**Results:**

Our results show that incubation of EA.hy926 cells with ET-1 led to a 6.5 ± 1.6 fold increase in IL-6 expression by qPCR, an event that was blocked by *S. jambos*. Also, we observed that ET-1 increased extracellular protein disulfide isomerase (PDI) activity that was likewise dose-dependently blocked by *S. jambos* (IC_50_ = 14 μg/mL). Consistent with these observations, ET-1 stimulated *ex vivo* human polymorphonuclear and mononuclear cell migration that also was dose-dependently blocked by *S. jambos*. In addition, ET-1 stimulation led to significant increases in ROS production that were sensitive to *S. jambos*.

**Conclusion:**

Our results suggest that the *S. jambos* extract represents a novel cardiovascular protective pharmacological approach to regulate endothelial cell activation, IL-6 expression, and immune-cell responses.

**Supplementary Information:**

The online version contains supplementary material available at 10.1186/s12906-022-03572-7.

## Background

Endothelial cells play an important role in maintaining vascular tone, platelet aggregation, cell adhesiveness, leukocyte migration, coagulation, and inflammation. Disordered endothelial cell activation contributes to the pathophysiology of diabetes [1], atherosclerosis, cancer, chronic inflammation, sepsis, and viral infections [2–5], such as observed in patients with the recently described Coronavirus Disease 2019 (COVID-19) [6, 7]. It is characterized, in part, by increased reactive oxygen species (ROS) and cytokine production, including endothelin-1 (ET-1) and interleukin-6 (IL-6), leading to apoptosis, increased leukocyte migration, accelerated endothelial cell senescence. These cytokines are important contributors to the cytokine release syndrome, also known as cytokine storm that is observed following viral infection, sepsis, and immunotherapy in cancer [8–12].

ET-1 is a 21-amino acid peptide and potent vasoconstrictor that acts via paracrine and autocrine mechanisms to increase vascular permeability leading to rapid and robust leukocyte recruitment [13]. ET-1 plays an important role in sepsis, cytokine release syndrome [12], and the cardiovascular complications of diabetes [14]. ET-1 induces oxidative stress in part by increasing vascular inflammatory responses, including increased IL-6 production [15, 16] and ROS generation resulting in vascular wall modifications [17, 18]. We reported that ET-1 also increases extracellular protein disulfide isomerase (PDI) activity and is secreted by endothelial cells [19]. PDI is an oxidoreductase enzyme, mostly known for its effects on platelet activation, including integrin-mediated platelet and leukocyte function in thrombogenesis [20, 21], cellular adhesion [22], nitric oxide (NO) delivery [23], leukocyte adherence [24] and oxidative stress sensor in endothelial cells [25]. Therefore, modulation of ET-1 levels and its effects are of great concern in cardiovascular diseases.

The pharmacological applicability of extracts from leaves of the *Syzygium jambos (S. jambos)* (L.) Alston tree, also known as *Eugenia jambos*, as a potential source of polyphenols for the treatment of cardiovascular disease, including hypertension, has been previously proposed [26]. *S. jambos* has been traditionally used to treat diabete [27]. *S. jambos* has anti-inflammatory activity [28, 29], antioxidant [30] and is reported to have antimicrobial properties as well [28, 31]. There are reports showing that various polyphenols have anti-inflammatory [32] and antioxidant properties [33, 34] and can prevent ET-1 effects [35]. However, in humans, the use of plant extracts is limited because of the absence of randomized controlled clinical trials due, in part, to the scarcity of information on the mechanisms by which polyphenols such as *S. jambos* mediate their effects. Our present study was designed to evaluate the effects of *S. jambos* on endothelial dysfunction. We hypothesized that the aqueous leave extracts from *S. jambos* would regulate ET-1-stimulated human endothelial cell function. We show that aqueous extracts from *S. jambos* inhibits PDI activity and decrease IL-6 expression, ROS production, and cell migration. This work describes novel and important effects of Syzygium jambos extract on endothelin-1 signaling and endothelial cell dysfunction.

## Materials and methods

### Materials

Folin–Ciocalteu reagent (Sigma-Aldrich, USA), gallic acid was purchased from ACROS Organics, EA.hy926 (ATCC, CRL-2922) were obtained from Manassas, VA. IL-6 ELISA kit from R&D Systems (Minneapolis, MN). QCM Chemotaxis 3-μm cell migration assay system was purchased from Millipore (Bedford, MA) and TRIzol reagent from Invitrogen (Thermo Fisher Scientific, USA). The Phusion reverse transcriptase PCR kit was obtained from New England Biolabs (Ipswich, MA). 5-(and-6)-chloromethyl-2′,7′-dichlorodihydrofluorescein diacetate, acetyl ester (CM-H2DCFDA) dye from Life Technologies, Trolox, Protein disulfide Isomerase from bovine liver, Rutin, bacitracin, Dithiothreitol (DTT), and all other reagents were purchased from Sigma-Aldrich (St. Louis, MO, USA).

### Plant collection, identification, and preparation

Leaves of *S. jambos* were collected in Puerto Rico in the town of Naguabo (18°14′29.2“ N and 65°45’30.8” W). Vouchers of *S. jambos* (019663) were numbered and deposited at the George Proctor Herbarium (SJ) in Puerto Rico. Dr. Robert Ross, Botanist from the University of Puerto Rico in Cayey, identified and classified the botanical species. Concoctions were prepared by boiling 30 g of plant material in 100 ml of distilled water. After concentration to 15 ml, the decoctions were filtered through a cheesecloth. Aliquots (5 ml) of the extracts were freeze-dried using a lyophilizer to determine the initial concentration of the rich-polyphenol extracts (μg/ml).

### Quality control of extract

The HPLC fingerprinting profile of plant extracts was carried out using a Shimadzu HPLC system with a Reverse Phase C18 column. The HPLC separations were conducted using an acetonitrile gradient with 0.5% acetic acid in water for 75 min with a 0.7 mL/min flow rate. The UV–VIS spectra (700–200 nm) were recorded for each extract using a UV-VIS spectrometer. The maxima absorption wavelengths were identified. The extraction factor (EF) of bioactive molecules from each extract were calculated to compare the yields of extraction in different batches, considering the absorption values (A) recorded for each λmax, multiplied with the dilution factor (d) and applying the following relation: EF = A (λmax)xd. This ensured that a similar amount of bioactive molecules were present in each experiment.

### Quantification of phenolic content

The total phenolic content was determined using a spectrophotometry method [36]. The *S. jambos* extract was prepared as a 1.0 mg/ml aqueous solution. Extract (0.5 ml) was mixed with 1 ml of Folin–Ciocalteu reagent (2 N) and was left to stand at room temperature for 1 min. The sodium carbonate (700 mM Na_2_CO_3_) solution (3 ml) was subsequently added, and the solution was allowed to stand at room temperature for 2 h. Supernatant absorbance was measured at 760 nm using a UV-VIS spectrometer. The results were compared with the standards prepared similarly (with known gallic acid concentrations). All samples were analyzed three times in separate experiments and were expressed as gallic acid equivalents in mg/g of dry extract.

### Quantification of flavonoid content

The total flavonoid content was determined using a colorimetric method [37]. The *S. jambos* extract was prepared as a 1.0 mg/ml aqueous solution. The extract (1 ml) was mixed with 0.4 ml of a 5% NaNO_2_ solution. The mixture was allowed to stay at room temperature for 6 min; 0.4 ml of a 10% AlCl_3_^*^9H_2_O solution was added for 6 min, followed by the addition of a 4 ml 4% NaOH solution. The ethanol solution (70%) was added to reach a final volume of 10 ml. The solution was mixed and kept at room temperature for 15 min. Absorbance was measured at 510 nm using a UV-VIS spectrometer. Comparisons were made against standards prepared similarly (with known rutin concentrations). Results were expressed as rutin mg/g of dry extract. All samples were analyzed three times in separate experiments.

### Cell culture

Human endothelial cells EA.hy926 (ATCC, CRL 2922) were maintained in DMEM (containing 4.5 g/L glucose) with 10% FBS as previously described by us [38]. Briefly, twelve hours before treatment, cells were serum-starved in DMEM with 0.2% FBS. At the treatment time, cells were washed with PBS and incubated with vehicle, ET-1, or *S. jambos* extract in DMEM with 0.2% FBS. The cells were harvested for analyses after 24-h or 48-h incubation.

### PDI. activity assays

#### Insulin turbidity assay

This assay is based on the measurement of the catalytic reduction of insulin as described by Lundstrom and Holmgren [39]. PDI facilitates the reduction of insulin in the presence of Dithiothreitol (DTT). The reduced insulin chains aggregate and the turbidity is monitored spectrophotometrically at 650 nm. The assay was performed in a 96-well plate format and a volume of 100 μl in the presence of 1 mM DTT, 1 μg PDI (Sigma), 0.15 mM bovine pancreas insulin (Sigma), and 0.2 mM EDTA in 100 mM potassium phosphate, pH 7.0. The progress of the reaction was monitored on a 96-well plate reader (Multiscan FC Microplate Reader, Fisher Scientific) at 650 nm for 60 min at 25 °C. The non-enzymatic reduction of insulin by DTT was recorded in control well without PDI.

#### Di-E-GSSG fluorescence assay

A soluble PDI activity assay was performed following previously described protocols and optimized in our laboratories [19]. Briefly, [E-GSH] formation is recorded for 30 min (*ex*: 525 nm; *em*: 545 nm) at room temperature, and fluorescence intensity is recorded and plotted as a function of time to calculate the conjugate’s reduction rate. Measurements were performed in the presence or absence of 16.5 ug/ml Phenylarsine oxide (PAO), a well-known vicinal thiol blocker. PBS was used as vehicle. Supernatant PDI activity was determined by fluorescence recording of [E-GSH] formation.

##### Quantitative real-time PCR

Total RNA was prepared with 1 mL of TRIzol reagent (Invitrogen) according to the manufacturer’s instructions. The Phusion reverse transcriptase PCR kit (New England Biolabs) was used to make 20 μL of cDNA from 1 μg of R.N.A. Gene expression was analyzed using real-time PCR using TaqMan gene expression assay for IL-6 and GAPDH (Applied Biosystems) in a StepOne Plus from ABI. The ΔΔ cycle threshold method was used to determine mRNA levels. IL-6 gene expression was normalized to GAPDH levels.

##### ELISA

To determine the concentration of IL-6 in cell culture media of control or treated endothelial cells, an ELISA kit from R&D Systems was used following the manufacturer’s instructions.

##### Cell migration assay

Cell migration was carried out using the QCM Chemotaxis 3-μm cell migration assay system (Millipore, Bedford, MA). *Ex vivo* human polymorphonuclear leukocytes (PMN) and mononuclear cells (MNC) from healthy human subjects were isolated using Polymorphprep following approval by the Brigham and Women’s Hospital– Institutional Review Board (Approval num. BWH IRB# 2009P000491) and written informed consent was provided to volunteers who participated in this study. Cells were seeded into the migration chamber as per the manufacturer’s protocol. The supernatant of endothelial cells was incubated for 2 h with the vehicle (PBS), ET-1, or *S. jambos* extract and placed in the lower chamber. After allowing cell migration for 2 h, PMN and MNC cells that migrated through the membrane were stained with Cyquant and quantified using Spectra Max Gemini EM (Molecular Devices) at 480/520 nm.

##### Measurements of Reactive Oxygen Species (ROS)

The **5-(and-6)-chloromethyl-2′,7′-dichlorodihydrofluorescein diacetate, acetyl ester** (CM-H2DCFDA) dye from Life Technologies was used as a probe to measure ROS. Endothelial cells were cultured in 6-well plates and were serum-starved for 12 h in DMEM with 0.2% FBS, washed with fresh DMEM, and incubated with DMEM premixed with 10 μM CM-H_2_DCFDA for 1 h at 37 °C. Cells were washed twice with PBS to remove the loading buffer, and cells were allowed to recover for 10 min at 37 °C for cellular esterases to deacetylate H_2_DCFDA. Cells were then stimulated with ET-1 with or without *S. jambos* extracts and Trolox (200 μM) for 1 h, releasing a variety of ROS species, which oxidize H_2_DCF into DCF Cells were washed once with Hank’s Balanced Salt Solution and incubated in the same buffer containing 5 μg of CM-H2DCFDA /ml at 37 °C for 30 min. Intracellular fluorescence was detected with excitation at 495 nm and emission at 520 nm using Spectra Max Gemini EM.

##### Annexin-V apoptosis assay

EA.hy926 cells were cultured in 96-well plates and were serum-starved for 12 h in DMEM with 0.2% FBS before the vehicle, ET-1, or plant extract was added for 24 h. The Annexin V FITC Assay Kit (Cayman Chemical) was used and analyzed using Spectra Max Gemini EM at 485/535 nm per manufacturer protocols to determine the percentage of apoptotic cells.

##### Statistical analysis

Data were analyzed by one-way or two-way ANOVA with Bonferroni post-test or Student’s *t-*test when appropriate. The *p*-value was set to be <0.05. Data are expressed as mean ± SE of (n) independent experiments unless otherwise stated.

## Results

### *S. jambos* inhibits ET-1 induced PDI activity

The concentration of polyphenols and flavonoids in *S. jambos* aqueous leaf extract was determined using spectrophotometric methods as described in methods. We determined that *S. jambos* aqueous leaf extract contains polyphenols 135 ± 7 Gallic acid mg/g and flavonoid 99 ± 9 rutin mg/g. Chagas et al. described the extract composition of Syzygium by HPLC-UV/Vis and LC-MS/MS using purified standards as calibrators at different wavelengths to validate its composition as myricetin as the most abundant flavonoid with gallic acid and quercentin [40] with high antioxidant potency. Eight peaks were identified in the *S. jambos* aqueous leaf extract HPLC fingerprinting used for quality control (Suppl Fig. 1).

**Fig. 1:**
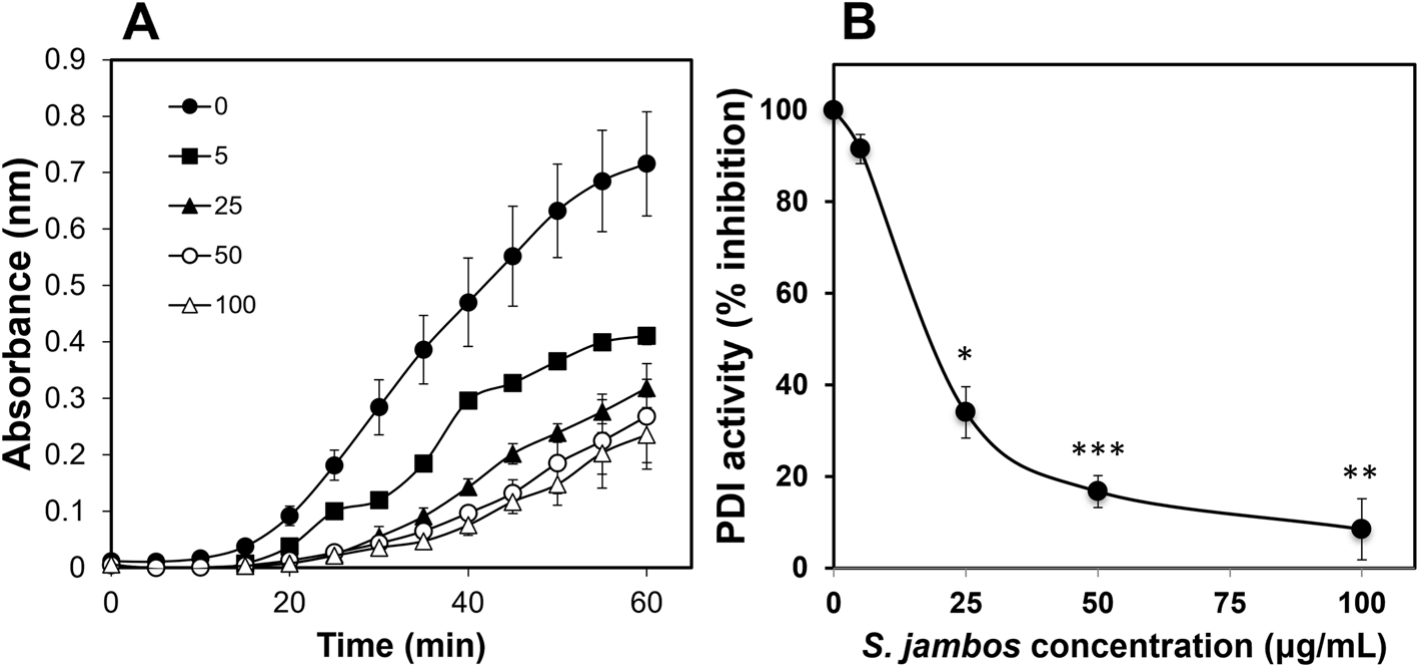
*S. jambos* inhibits PDI activity. PDI Insulin turbidity assay was performed in the presence of increasing concentrations of *S. jambos* extract (0–100 mg/ml) with 5 μM purified PDI as described in *Methods*. A) PDI activity in presence and abschence of *S. jambos* extract. B) PDI activity was inhibited by *S. jambos* extract in a dose-dependent manner with an IC_50_ of 14 μg/mL. Values represent mean ± SE of 3 experiments in duplicate determinations. *p < 0.011,**p < 0.005, ***p < 0.004 when compared to control

One of the effects of S. jambos that has a lot of interest was its effects on protein disulfide isomerase (PDI). It has been shown that myricetin [41] and quercentin [20] inhibit the reductase activity of PDI. As the major components of the S. jambos, we evaluated the effect of extract preparation on PDI activity. We measured *in vitro* PDI activity in the presence or absence of the *S. jambos* aqueous leaf extract using the insulin turbidity assay. It shows a significant reduction of 5 μg of purified PDI activity with 5 μg/mL and up to 75% inhibition at 100 μg/mL of *S. jambos* extract (*p* = 0.004, *n* = 3) (Fig. 1A). We also observed that *S. jambos* extract induced a dose-dependent inhibitory effect on purified PDI with an IC_50_ of 14 μg/mL (Fig. 1B).

**Fig. 2:**
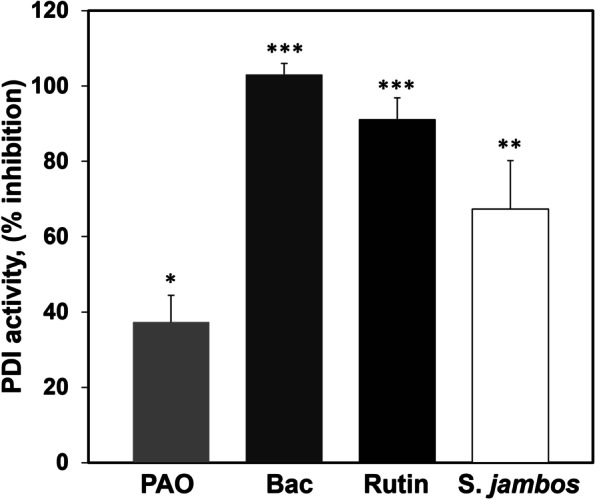
*S. jambos* is an inhibitor of PDI activity. Turbidity assay was used to measure the degree of inhibition of *S. jambos* extracts (50 mg/ml) compared to well-known PDI inhibitors at maximal concentrations. Phenylarsine oxide (PAO,16.8 μg/ml), bacitracin (Bac, 1.4 mg/ml), and Rutin (0.6 μg/ml) in the presence of 5 μg purified PDI. PBS was used as vehicle for 100% PDI activity. Values represent mean ± SE of 5 experiments in duplicates. *p < 0.01, **p < 0.002, *** *p* < 0.0001

The effectiveness of *S. jambos* on PDI reductive capacity inhibition compared to other well-known inhibitors was examined *in vitro* [42–44]. These agents’ inhibitory mechanisms are not well known, but it involves covalent binding of an open thiol site to free cysteines in the substrate-binding domain of PDI. In vitro assayed of the extract against phenylarsine oxide, PAO (16.8 ug/mL), bacitracin (1.4 mg/mL) and Rutin (quercetin-3-O-rutinoside, 0.6 ug/mL) is represented in Fig. 2. It shows that 50 μg/mL of *S. jambos* inhibits purified PDI reductive capacity by 67% but is less effective than Rutin (citrus flavonoid) or bacitracin (antibiotic) at maximal inhibitory concentrations. Previous extractions of S. jambos were not tested on purified PDI activity to assess its crude effective capacity, but *in vivo* platelets activation, the extract was able to block approximately 60% of ADP-activated platelets [41].

**Fig. 3:**
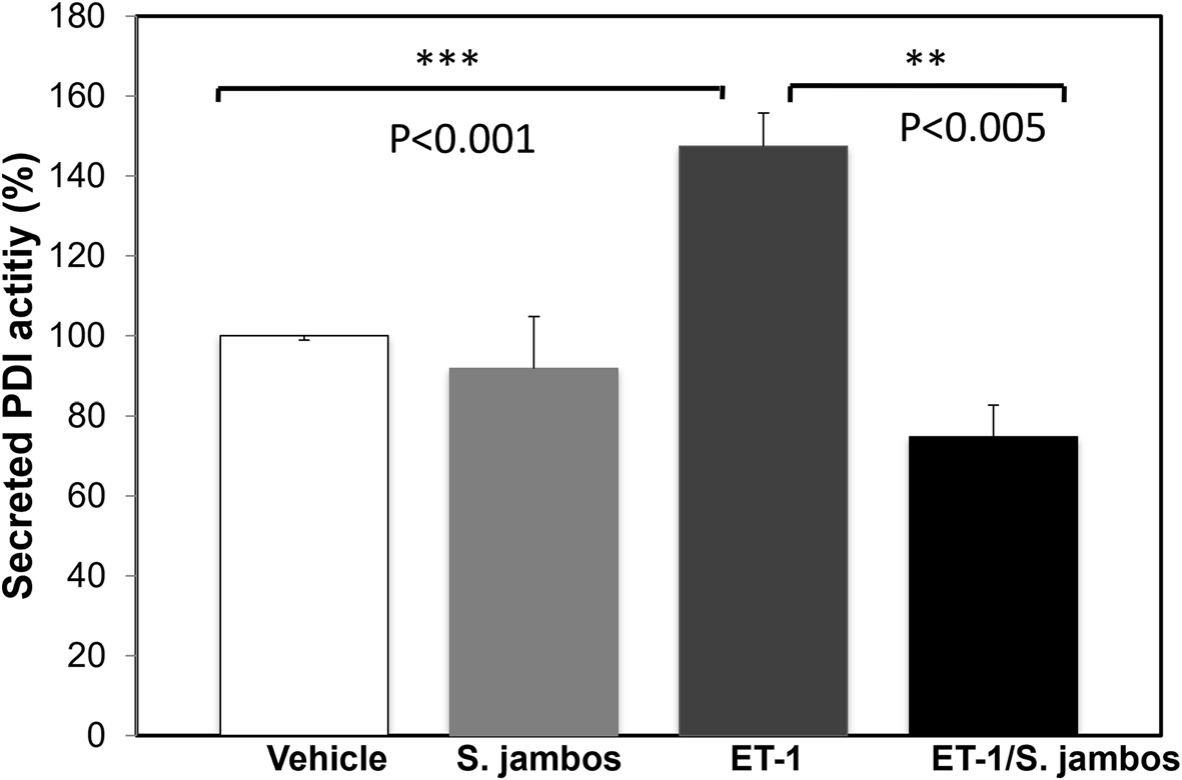
*S. jambos* blocks ET-1 -stimulated PDI activity in human endothelial cells. Human endothelial cells were incubated in the presence or absence of 100 nM ET-1 with or without *S. jambos* extract (50 mg/mL). PDI activity was measured in the supernatants after 2 h incubation at 37 °C and expressed as % of the activity described in *Methods*. Values represent mean ± SD (*n* = 6). **p < 0.005, *** *p* < 0.001

We previously demonstrated that ET-1 induced PDI activity in endothelial cells [19] as well as in erythrocytes [45]. To test whether the extract *S. jambos* can inhibit endothelin-1 (ET-1)-stimulated PDI activity in human endothelial cells, we measured the effect of 50 μg/mL *S. jambos* on cell-secreted PDI activity using a sensitive fluorescence assay as described in methods (Fig. 3). It showed that ET-1 induced a significant increase in cell-secreted PDI (*p* = 0.001, *n* = 6) that was completely blunted by the presence of *S. jambos* (*p* < 0.005, *n* = 6).

### S. jambos inhibits IL-6 gene expression and secretion

**Fig. 4:**
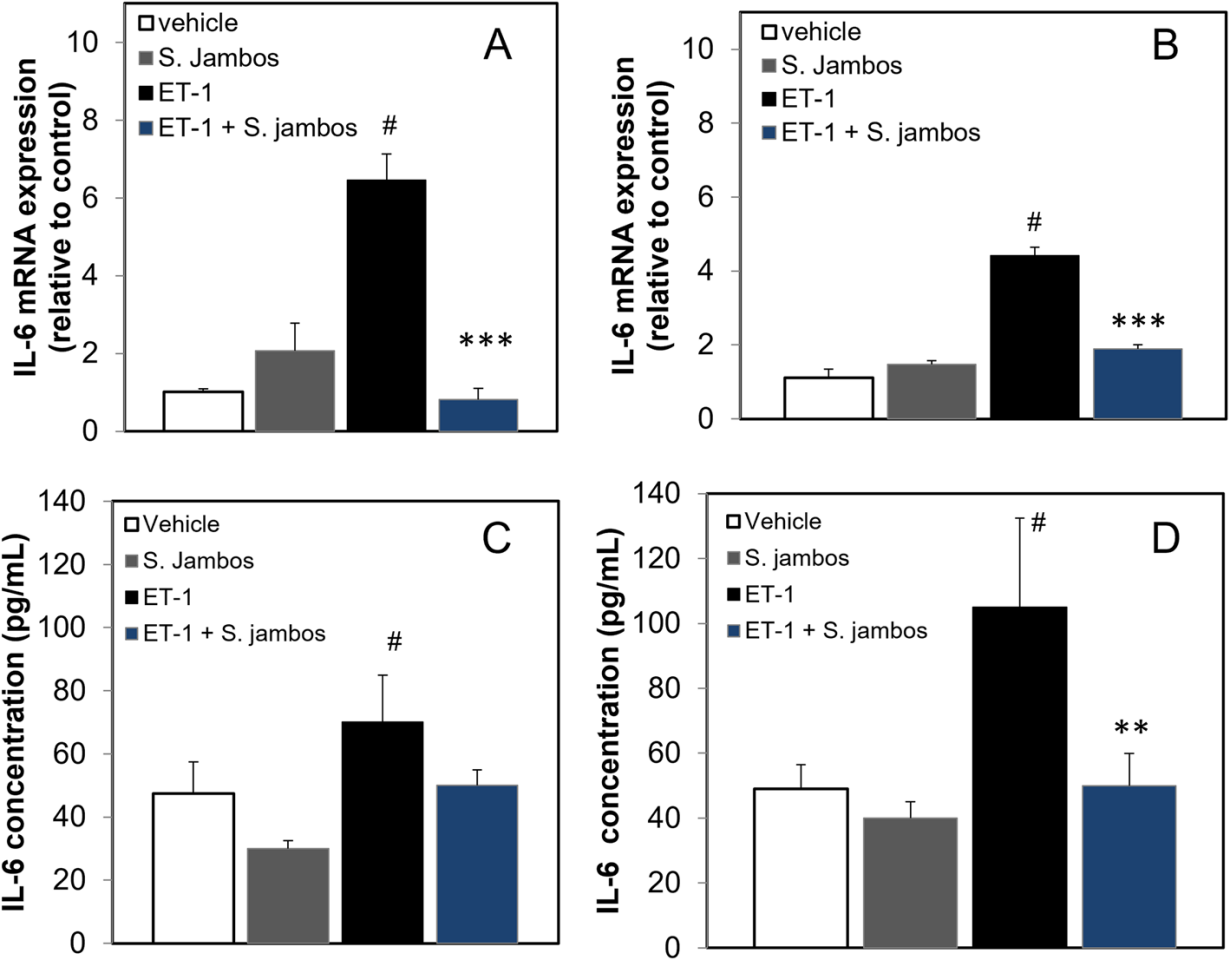
*S. jambos* extract downregulates the ET-1 stimulated IL-6 expression and secretion in endothelial cells. Human endothelial cells were incubated for 24 (**A**, **C**) or 48 h (**B**, **D**) in the absence or presence of 100 nM ET-1 with or without *S. jambos* extract (50 μg/ml). mRNA levels are presented as the fold difference in RNA level relative to unstimulated sample values using GAPDH as a control. **A-B** mRNA levels of IL-6 by real-time PCR was measured as described in *Methods* at 24 h (A) #*p* < 0.0002 vehicle vs ET-1; ***p < 0.0008, ET-1 vs ET-1 + S. *jambos* and 48 h (B) #*p* < 0.0003 vehicle vs ET-1; ***p < 0.0001, ET-1 vs ET-1 + S. *jambos*. Values represent mean ± SE (*n* = 6). **C-D** Supernatants were collected to determine secretion levels of IL-6 as measured by ELISA assay as described in Methods. Results represent the mean ± SD of *n* = 3. **, *p* < 0.01 ET-1 treated samples in absence vs presence of *S. jambos* extract; #*p* < 0.001 vehicle vs ET-1

Mounts of evidence demonstrated that ET-1 effects on the endothelium are part of the inflammatory process. To evaluate the effects of *S. jambos* on the inflammatory cascade, EA.hy926 cells were treated with 100 nM of ET-1 in the presence or absence of 50 μg/mL of *S. jambos* for 24 and 48 h (Fig. 4A-B). We observed that ET-1 significantly upregulates IL-6 mRNA expression by 6.5 ± 1.6 folds (*p* = 0.0002, *n* = 6), and S. jambos significantly inhibited this upregulation (0.82 ± 0.73 folds, *p* < 0.001, *n* = 3) (Fig. 4A). We also observed that IL-6 mRNA expression upregulation by ET-1 is optimal 24 h after stimulation (Fig. 4B). We also observed that *S. jambos* extract alone had no significant effect on the expression of IL-6 (Fig. 4 A-B), suggesting that the extract bioactive agents interfere with the ET-1 signaling pathway and not the direct IL-6 synthesis.

IL-6 protein secretion was also measured by ELISA to assess whether *S. jambos* extract directly inhibits its release. EA.hy926 cells were treated with 100 nM ET-1 with and without 50 μg/mL *S. jambos* for 24 or 48 h as described in Fig. 4A-B. We found that *S. jambos* inhibited ET-1-induced secretion of IL-6 (*p* < 0.01, *n* = 3) (Fig. 4C). In addition, we observed S. jambos extract was able to significantly inhibit ET-1 induced IL-6 expression even though the ET-1 effects were declining 48 h later (*p* < 0.001, *n* = 3) (Fig. 4B). This effect was reflected at the synthesis level which we observed a strong decrease in IL-6 secretion after 48 h of incubation with the extract (Fig. 4D).

### S. jambos blocks leukocytes migration

**Fig. 5:**
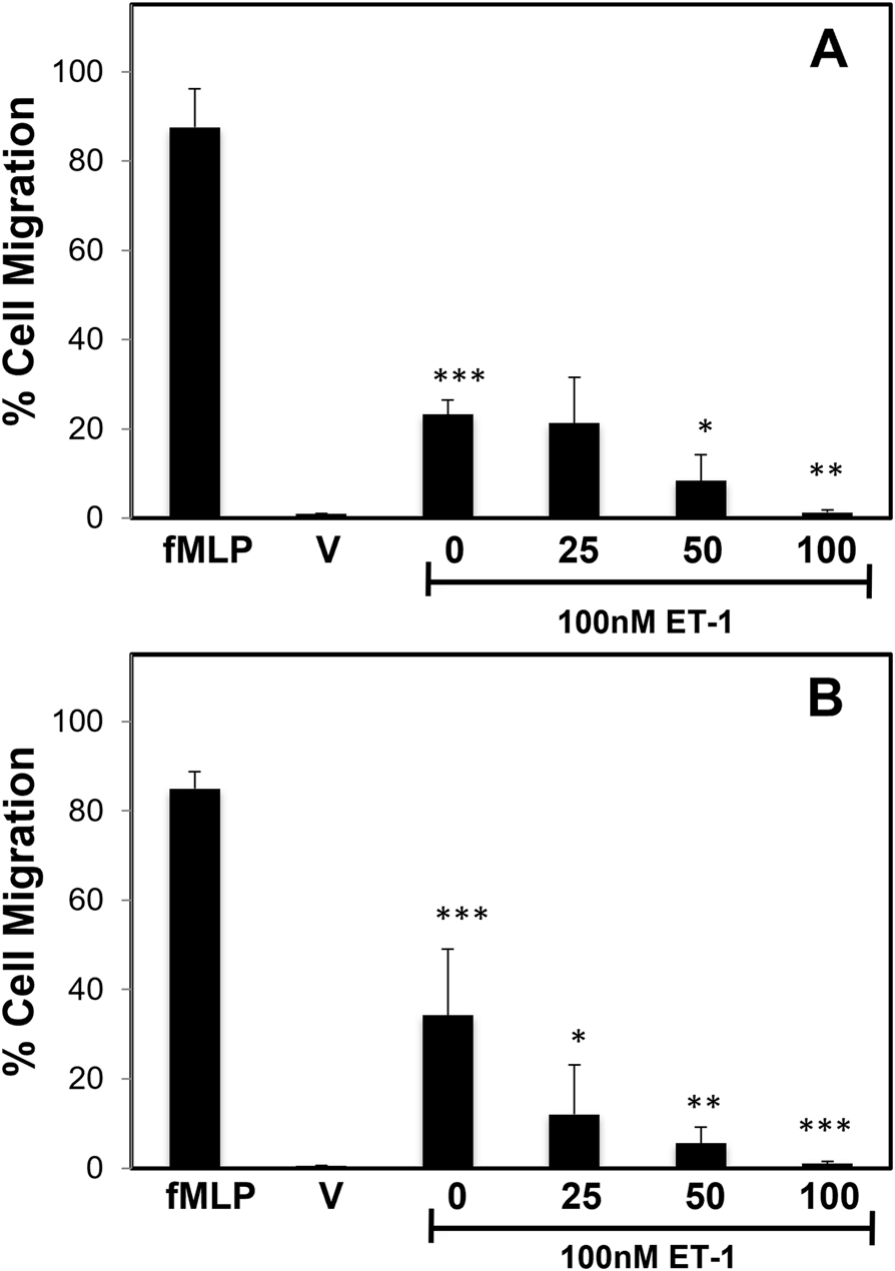
*S. Jambos* extract regulates Leukocyte migration on ET-1 stimulated endothelial cells. Human endothelial cells were cultured for 2 h with 100 nM ET-1 alone (with PBS as vehicle) or in the presence of varying concentrations of *S. jambos* extract (from 25 to 100 μg/mL) and the supernatant collected. *Ex vivo* PMN (**A**) and MNC (**B**) from healthy human volunteers were placed in an upper chamber as described in Methods. fMLP (1 μM), a potent chemoattractant, was used as a positive control. PBS was used as vehicle (V). Results represent the mean ± SD of *n* = 3. ET-1 treated samples in the absence vs presence of *S. jambos* extract *, *p* < 0.05 **, *p* < 0.01 ***, *p* < 0.001

The S. jambos leaf oil extract has shown an inhibitory effect on lipopolysaccharide (LPS)-induced migration of eosinophils and neutrophils [46]. To study the effect of *S. jambos* on endothelin-induced leukocyte migration, we characterized the *ex vivo* effects of ET-1-stimulated cells human polymorphonuclear (PMN) cells and mononuclear (MNC). Freshly isolated leukocytes were incubated in the supernatant of activated endothelial cells with ET-1 for 1 h in the presence or absence of *S. jambos*. ET-1 significantly induced the migration of PMN (*n* = 3, *p* < 0.001; Fig. 5A) and MNC (*n* = 3, *p* < 0.001; Fig. 5B). The chemotactic agent, N-Formyl-Met-Leu-Phe (fMLP), was used as a positive control for these experiments. We observed PMNC and MNC migration was significantly inhibited by the presence of *S. jambos* in a dose-dependent manner (Fig. 5 A-B). The extract completely blunted the effect of ET-1 cellular migration at 100 μg/mL in both cell types.

### *S. jambos* inhibits ROS production in endothelial cells

**Fig. 6:**
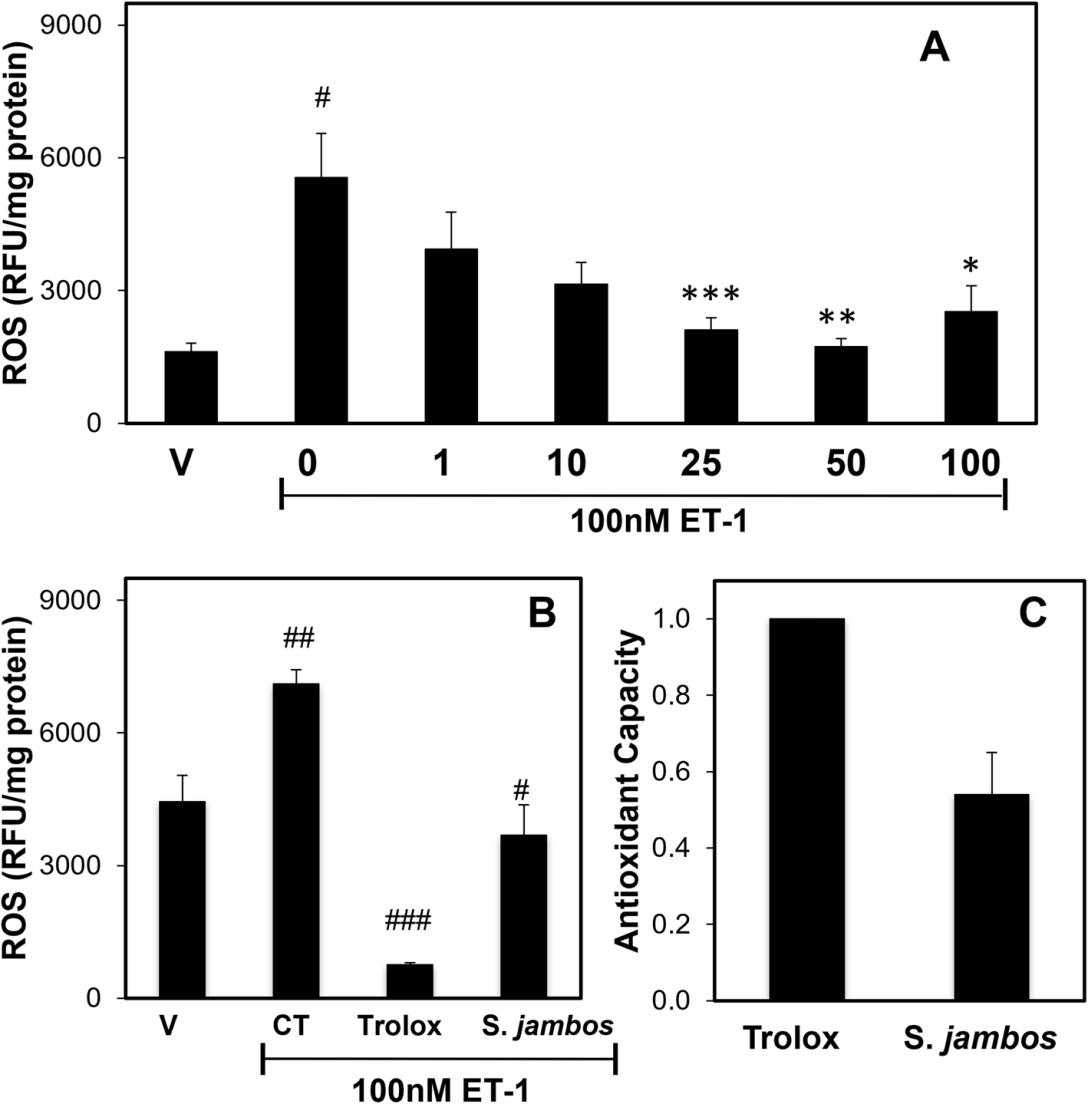
*S. jambos* extract regulates the ET-1 stimulated ROS production in endothelial cells. **A** Human endothelial cells were incubated for 1 h with different concentrations (from 1 to100 μg/ml) of *S. jambos* extract or PBS as vehicle (0 μg/ml *S. jambos*) followed by 100 nM ET-1 stimulation for 1 h. ROS was determined using CM-H2DCFDA fluorescence as described in methods. Values represent the mean ± SD of *n* = 4. *, *p* < 0.02; **, *p* < 0.004 ***, *p* < 0.009. ET-1 treated samples in absence vs. presence of *S. jambos* extract. ^#^*p* < 0.001 vehicle vs ET-1. **B-C** comparison between Trolox (200 μM) and S. *jambos* (50 μM/mL) ROS inhibitory capacity. To determine the antioxidant capacity of *S. jambos* extract, we used Trolox as a positive control. Trolox equivalent antioxidant capacity (TEAC) for *S. jambos* extract was 54%. Values represent the mean ± SE (*n* = 5). #*p* < 0.001, ###*p* < 0.00002, Trolox vs S. *jambos* in the present of ET-1. ##*p* < 0.001, vehicle vs ET-1

Previous studies indicated that S. jambos extract plays a role in ROS regulation; however, contradictory evidence shows an increase in ROS production, leading to apoptosis [47] or a decrease in ROS, reducing glucose-induced stress [48]. Figure 6 shows ROS levels in EA.hy926 cells using fluorescent dye CM-H2DCFDA as described in Methods to evaluate if S. jambos extract can regulate ET-1 induced ROS production. We observed that pre-incubation of endothelial cells with various concentrations of S. jambos significantly blunted the stimulation of ET-1-induced R.O.S. production by 2-folds compared to vehicle (Fig. 6A, *n* = 4). We also observed that *S. jambos* extract blocked the ET-1 stimulated ROS production as low as 25 μg/mL (*p* < 0.01, *n* = 4) (Fig. 6A). To determine the antioxidant potential of *S. jambos* extract, we compared the effect of 50 μg/mL *S. jambos* with 200 μM Trolox (500 μg/mL), a known powerful scavenger of intracellular ROS. As expected, Trolox completely inhibited ET-1 induced ROS activity (Fig. 6B). We also observed that S. *jambos* shared a similar function as Trolox, suggesting its strong capacity as an antioxidant with a Trolox equivalent antioxidant capacity of 54% (*n* = 3) (Fig. 6C).

### *S. jambos* reduces the apoptotic effects of ET-1 stimulated endothelial cells

**Fig. 7:**
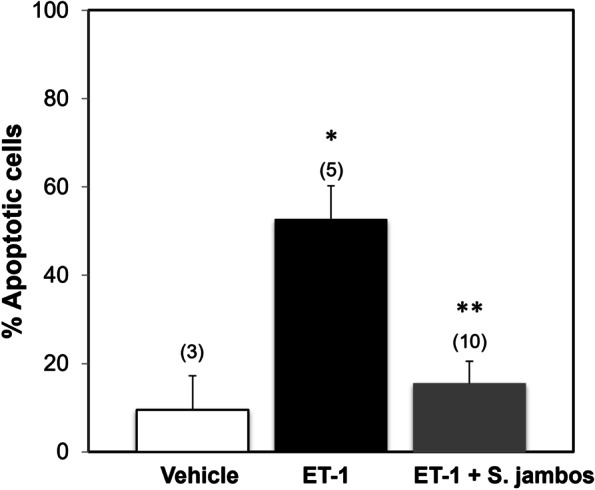
*S. Jambos* extract regulates the apoptotic activity of ET-1 stimulated endothelial cells. Human endothelial cells were incubated for 24 h in the absence or presence of 100 nM ET-1, with or without 50 μg/mL *S. jambos* extract. Apoptosis was determined using the Annexin V dye as described in the method. Results are presented relative to dry cells, which we considered as 100% apoptosis per manufacture protocols. PBS was used as vehicle. Results represent the mean ± SE. (n) in parenthesis. ET-1 vs Vehicle *p < 0.003; ET-1 vs ET-1 + *S. jambos* extract **p < 0.0006

ET-1 has been shown to induce apoptotic activity in endothelial cells [49]. To evaluate *S. jambos* extract on ET-1 induced apoptotic effect in endothelial cells, we used Annexin-V Apoptosis Assay (Cayman Chemical) as a measuring tool (Fig. 7). As observed, we found that treatment of endothelial cells with ET-1 for 24 h significantly increased endothelial cells apoptosis (Fig. 7). This effect was significantly blocked by the presence of *S. jambos* extract by 55%.

## Discussion

Disordered levels of ET-1 and endothelial cell activation play essential roles in endothelial dysfunction and the worsening of vascular complications in diabetes, cardiovascular diseases, and sepsis. As such, it remains an important area of research. This study investigated the effects of *S. jambos* aqueous leaves extract on ET-1-mediated endothelial cell activation regulation. We showed that the *S. jambos* extract significantly inhibits purified PDI activity *in vitro* with a potency comparable to Rutin, a well-known specific inhibitor of PDI activity [20]. We also observed that the *S. jambos* extract had anti-inflammatory and anti-oxidative properties as it reduced IL-6 secretion/expression, leukocyte migration, and ROS production from ET-1 stimulated endothelial cells. These results suggest that the *S. jambos* extract represents a novel modulator of ET-1 regulated endothelial cell activation and inflammatory responses. As a regulator of ET-1 mediated endothelial cell and leukocyte function, our data support the contention that *extracts from the S. jambos leaves* may have medicinal benefits that could be studied in rodent models of cardiometabolic disease.

*S. jambos* extracts showed a potent effect on ET-1 mediated leukocyte migration. These results are important as ET-1-stimulated IL-6 gene expression has been implicated as an important and independent regulator of endothelial cell activation [50] as it likewise increased leukocyte recruitment to the endothelium [51]. In addition, IL-6 has been reported to play an important role in response to infection, hematopoiesis, and immune activation [52]. In contrast to what was reported with quercetin effect on IL-6 synthesis [53], we found no effect of S. jambos (high content of quercetin) on IL-6 expression or secretion, suggesting that ET-1 stimulated IL-6 was mediated by blockade of the ET-1 signaling pathway.

Excess IL-6 levels also stimulate angiogenesis and increased vascular permeability via increased VEGF levels, as observed in rheumatoid arthritis [54]. Our data suggest that *S. jambos* may serve as an alternative or adjuvant therapeutic approach to control cytokine-induced IL-6 secretion and endothelial cell activation. Our report expands and extends the growing amount of data showing that phytochemicals, such as phenolic compounds in medicinal plants, are associated with increased anti-inflammatory and antioxidant capacity [55, 56]. Our data support the contention that *extracts from the S. jambos leaves* may have medicinal benefits due to their anti-inflammatory [28, 29], antioxidant properties [30], and as a regulator of ET-1 mediated endothelial cell function.

ET-1 stimulates ROS production, primarily superoxide anions (O_2_^−^), leading to oxidative stress. We observed that *S. jambos* extract abrogated ET-1 stimulated ROS production in endothelial cells demonstrating that this extract has the capacity of reducing harmful oxidant radicals generated following endothelial cell activation. ET-1 mediates its effects through ETA and ETB receptors. Both receptors have been shown to regulate ROS production [49, 57]. ET-1 activates these receptors to mediate potent vascular contraction, cellular proliferation, and a pro-inflammatory effect. At high intravenous levels, ET-1 causes vasodilation and chronic contractions resulting in organ ischemia and endothelial dysfunction [58]. Under these conditions, ROS generation is elevated [35, 59] and has been proposed to induce lipid peroxidation and reduction of glutathione and SH groups [60]. ET-1 receptor blockade has been shown to restore total glutathione and superoxide dismutase activity [61, 62]. Interaction of ROS with glutathione (GSH) and thiols proteins determines cellular redox homeostasis and maintenance [63]. The potent effects on ROS production that we observed with the extract would suggest that the extract may also block the effects of ET-1 by increasing glutathione (GSH) availability and reducing ROS in addition to its anti-inflammatory actions. GSH plays an important role in the redox regulation of transcription factors and enzymes for signal transduction as an endogenous antioxidant. Indeed, recently it has been shown that flavonoids modulate the expression of gamma-glutamylcysteine synthetase, the rate-limiting enzyme in the synthesis of GSH [64]. The authors also demonstrated that flavonoids increase the expression of gamma-glutamylcysteine synthetase and, with it, an increase in intracellular GSH. Thus, we posit that in addition to the potential radical scavenging abilities of *S. jambos*, this extract also may be important in regulating the procoagulant and pro-inflammatory responses at sites of vascular injury.

In this study, we show that *S. jambos* regulates PDI activity. PDI is a multifunctional protein that provides essential isomerase and chaperone activities in the endoplasmic reticulum and the plasma membrane [65]. This ubiquitous protein introduces disulfides into proteins and catalyzes the rearrangements of correct disulfides. There is mounting evidence that PDI is involved in many physiological processes that play a major role in inflammatory processes and oxidation-reduction reactions essential for survival. We previously demonstrated that selective blockade of ET-1 receptors decreases PDI secretion in a mouse model of sickle cell disease [19] while improving the inflammatory and erythrocyte dehydration status, suggesting PDI as a therapeutic target in hematological and vascular diseases. Consequently, there is growing interest in developing novel therapeutics that can regulate ET-1 signaling pathways and PDI activity in various atherothrombotic, vascular and hematological diseases [66, 67]. Currently available selective and nonselective PDI inhibitors that have been used in characterizing PDI function include: Anti-PDI antibodies [RL90 and RL77] [68, 69] (ab5484 & ab2792 from Abcam); Phenyl arsine oxide (PAO); bacitracin [41]; N-Oxalylglycine; 16F16A; and the more recently described flavonoid, Rutin [70].

## Conclusion

In conclusion, the present in vitro study results show that the aqueous extract of S. jambos leaves blocks secreted PDI activity and IL-6 expression, which then regulates the antioxidant and anti-inflammatory of activated human endothelial and immune cells. These studies suggest that *S. jambos* polyphenols act as novel regulators of ET-1 activation pathways and strengthen the confirmation of the pharmacological benefits of the phytochemicals present in S. jambos to applications in the management of inflammatory disorders and endothelium dysfunction disorders.

## Supplementary Information

**Additional file 1: Supplementary Figure 1.** Typical fingerprinting chromatogram of *S. jambos* extract. Eight peaks were selected for the quality control of the S. jambos extract. Gallic acid and Rutin were identified using retention times of standards.

## Data Availability

The datasets generated and analysed during the current study are available in Mendeley Data, 10.17632/92ykngb24t.1.

## Abbreviations

ROS: Reactive oxygen species
ET-1: Endothelin-1
PDI: Protein disulfide isomerase
NO: Nitric oxide
IL-6: Interleukin-6
PAO: Phenylarsine oxide
*S. jambos*: *Syzygium jambos* (L.) Alston
DTT: Dithiothreitol
EF: Extraction factor
PMN: Polymorphonuclear leukocytes
MNC: Mononuclear cells
fMLP: N-Formylmethionyl-leucyl-phenylalanine
