# Supplementary material for: Inhibitory effects of Syzygium jambos extract on biomarkers of endothelial cell activation
Source: BMC Complement Med Ther. 2022 Apr 7;22:101. doi: 10.1186/s12906-022-03572-7 (PMC8988540; doi:10.1186/s12906-022-03572-7)
Supplement: Supplementary file 1 — Additional file 1: Supplementary Figure 1. Typical fingerprinting chromatogram of S. jambos extract. Eight peaks were selected for the quality control of the S. jambos extract. Gallic acid and Rutin were identified using retention times of standards. [file 12906_2022_3572_MOESM1_ESM.docx]

**
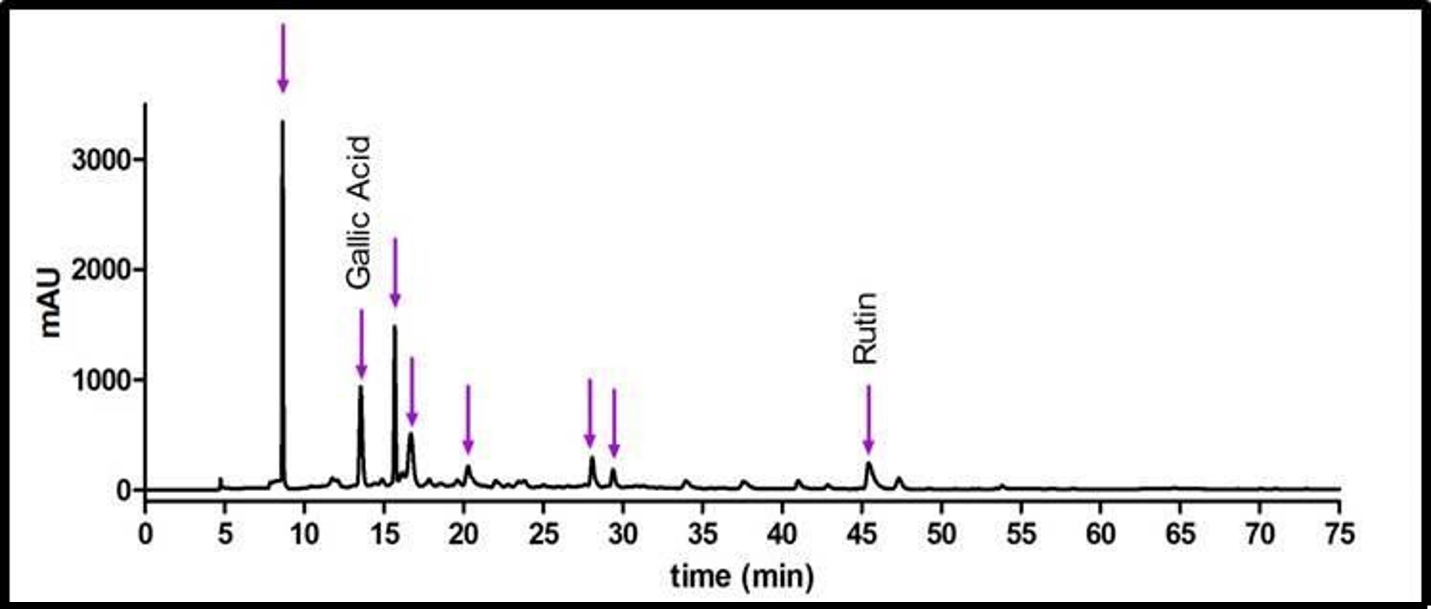
**

**Supplementary Figure 1. Typical fingerprinting chromatogram of *S. jambos* extract.** Eight peaks were selected for the quality control of the S. jambos extract. Gallic acid and Rutin were identified using retention times of standards.
